# Structural dualities between the Schrödinger equation and its ultra-slow-light counterpart in one spatial and one temporal dimension

**DOI:** 10.1038/s41598-026-42922-0

**Published:** 2026-03-17

**Authors:** José Rojas, Enrique Casanova, Melvin Arias

**Affiliations:** 1 Instituto de Física, Universidad Autónoma de Santo Domingo, Av. Alma Mater, 10105 Santo Domingo, Dominican Republic; 2 Laboratorio de Nanotecnología, Área de Ciencias Básicas y Ambientales, Instituto Tecnológico de Santo Domingo, Av. Los Próceres, 10602 Santo Domingo, Dominican Republic

**Keywords:** Mathematics and computing, Physics

## Abstract

Extreme kinematic limits of space-time symmetries reveal alternative forms of quantum behaviour. In the Carrollian limit the speed of light is taken to zero ($c \rightarrow 0$): causal light cones collapse onto the time axis, spatial points become disconnected, and the usual notion of particle motion disappears. The corresponding quantum equation is the Carroll–Schrödinger equation—the structural mirror image of the ordinary Schrödinger equation, being first order in space and second order in time. We explore the mathematical connections between these two equations in one space and one time dimension. Using operator methods, we find conditions on external potentials that allow both equations to share solutions, and show that any Carrollian problem can be mapped to an equivalent Schrödinger problem via an explicit coordinate transformation. Probability densities and currents are related by removing the interaction potential and swapping space and time coordinates. An extreme relativistic boost combined with a classical approximation yields the Carrollian dispersion relation and conditions for coinciding classical trajectories. By formulating the dynamics on a Hilbert space of time rather than space, we prove that evolution in the spatial coordinate preserves probability. Closed-form solutions illustrate each result and together offer a practical framework for translating between the two descriptions.

## Introduction

The Schrödinger equation admits several complementary derivations; one standard route is a symmetry construction using (projective/unitary) realizations of Galilean or Schrödinger invariance^1–3^, alongside canonical (e.g. Dirac; Sakurai–Napolitano)^4,5^, spectral/operator–theoretic^6,7^, and path–integral approaches^8,9^. Exploring the kinematic extremes of space-time symmetries reveals distinct physical regimes. While the Galilean limit corresponds to an infinite speed of light ($c \rightarrow \infty$), the opposite extreme is the ultra-relativistic “Carrollian” limit. Originally named by Lévy-Leblond^10^ in reference to the stationary world of Lewis Carroll, this regime arises when the speed of light is mathematically taken to zero ($c \rightarrow 0$). In this limit, standard causal light cones collapse around the time axis, causing strictly spatial points to become causally disconnected; classical motion ceases, and evolution is entirely temporal.

Carrollian symmetry has become increasingly central in modern discussions, with applications ranging from gravity and flat-space holography to dark-energy motivated settings (e.g.^11–31^). On the quantum side, adapting the dynamics of particles to this extreme kinematic zero-speed-of-light regime yields the *Carroll–Schrödinger* equation. This equation can be rigorously obtained as an ultra–relativistic limit of the Klein–Gordon equation^32^, and it admits a natural interpretation within the post–Carrollian^33^ viewpoint. In this framework, it emerges directly from the post–Carrollian energy–momentum relations and the corresponding operator structures^34^:

1 $$\begin{aligned} i\hbar c\,\partial _x \Psi (x,t)\;+\;\frac{\hbar ^2}{2m c^2}\,\partial _t^2 \Psi (x,t)\;=\;0, \end{aligned}$$

which is first order in space and second order in time. In $1+1$ dimensions, the free Post–Carroll structure is mathematically equivalent to the standard Schrödinger structure under the coordinate inversion $x \leftrightarrow ct$, reflecting the isomorphism between the corresponding symmetry groups in $1+1$ D^34^. We therefore focus on the nontrivial content that emerges once interactions are admitted, and we develop an operator-level dictionary that tracks how potentials, solutions, conserved currents, inner products, and classical limits translate between the two formalisms. We begin by encoding the dynamics with differential operators $\hat{\mathscr {H}}$ (Schrödinger) and $\hat{\mathscr {F}}$ (Post–Carroll) and identifying a differential operators compatibility criterion (namely, that the constraint class $\ker \hat{\mathscr {F}}$ is preserved by the Schrödinger evolution and vice versa) as genuine commutation $[\hat{\mathscr {H}},\hat{\mathscr {F}}]=0$. This constrains the relationship between the spatiotemporal Schrödinger potential $V_{sch}(x,t)$ and the Post–Carrollian spatiotemporal potential $V_{car}(x,t)$. Next, we construct a potential–dependent reparametrization $x=\delta (t)$ that maps the space–independent Post–Carroll problem to the time–independent Schrödinger equation and derive closed formulas for the dual $V_\textrm{sch}(x)$ in terms of $V_\textrm{car}(t)$. Eliminating $V_\textrm{car}$ yields a Schwarzian relation—a differential condition on the map $\delta$ determined by the curvature of the reparametrization—that we invert explicitly to specify $\delta$ for any static $V_\textrm{sch}$; harmonic, Coulomb–like, and free examples illustrate the dictionary. Conserved structures are then related by a gauge removal of $V_{car}$ and a coordinate inversion $(x,ct)\mapsto (ct,x)$, which identifies the Post–Carrollian continuity equation with the Schrödinger one, as expected from the $1+1$ D isomorphism between the groups under the coordinate inversion, as also outlined in^34^. We connect to relativistic and classical structures: we derive a Post–Carrollian dispersion relation by ultra–boosting the energy–momentum two–vector, providing a direct route from Lorentz kinematics to the Post–Carroll sector. We also work out the classical limit of the Carroll–Schrödinger dynamics via the Hamilton–Jacobi formalism, explore the fundamental differences with the Newtonian analog and a relationship between the spatial potentials that ensures classical particles follow the same trajectories in the $x$–$t$ plane. Post–Carroll dynamics is then placed on the equal–*x* Hilbert space $L^{2}(\mathbb {R}_t)$; we identify the self-adjoint *x*-evolution generator, determine its domain, and verify unitarity. We also explore the dynamics of Carroll–Schrödinger solutions by analyzing exactly solvable models, including a closed-form Gaussian packet and *finite–time quantization* for time-localized interactions. For general $V_{car}(x,t)$, a unitary gauge reduction produces an *interaction momentum* $F(x,t)=\,\partial _x\!\int ^{t}\!V(x,\tau )\,d\tau$ and recasts the problem as Schrödinger–type evolution in *x* with the minimal substitution $p\mapsto p-F$; we develop a controlled Dyson expansion in the spatial evolution variable and discuss the structural similarity of the gauge-transformed equation to the equations describing the evolution of temporal solitons and Cherenkov radiation in soliton media^35,36^. *Guide to the paper.* Section “Shared solutions and compatibility conditions” formulates the shared–solutions conditions and fixes the admissible potentials. Section “From the time-independent Schrödinger equation to the space-independent Carroll–Schrödinger equation” builds the map $x=\delta (t)$, derives the Schwarzian relation, and presents solvable examples. Section “Probability currents and densities” relates currents and densities between both formalisms. Sections “From Schrödinger to Carroll–Schrödinger in d=1+1 via a v$\rightarrow \infty$ boost” and “Hamilton–Jacobi limit” derive the Post–Carrollian energy–momentum relation via an ultra–boost and obtain the Hamilton–Jacobi classical limit from the Carroll–Schrödinger equation. Section “Carroll–Schrödinger inner product and generators” introduces the Post–Carrollian inner product and establishes the equal–Hamilton–Jacobi limit*x* Hilbert–space picture. Section “Solutions analysis” analyzes the behavior of exact solutions of the Carroll–Schrödinger equation, outlining two important cases (Gaussian correspondence and finite–time quantization). It also outlines a general solution method for a given potential $V_{car}(x,t)$ via a unitary gauge reduction of the equation and the introduction of an effective interaction–momentum and a spatial Dyson expansion, with physical interpretations.

## Shared solutions and compatibility conditions

Merging the Schrödinger and Carroll–Schrödinger equations with a potential,

2 $$\begin{aligned} & -\frac{\hbar ^2}{2m}\,\frac{\partial ^2 \Psi }{\partial x^2} + V_{sch}(x,t)\,\Psi - i\hbar \,\frac{\partial \Psi }{\partial t} = 0, \end{aligned}$$

3 $$\begin{aligned} & i\hbar c\,\frac{\partial \Psi }{\partial x} \;-\; \frac{1}{2m c^2}\,\Big (-i\hbar \,\frac{\partial }{\partial t} - V_{car}(x,t)\Big )^{2}\Psi = 0. \end{aligned}$$

Defining the operators

4 $$\begin{aligned} \hat{\mathscr {H}}=\frac{\hat{p}^{\,2}}{2m}+\hat{V}_{sch}(x,t)-\hat{E}, \qquad \hat{\mathscr {F}}=c\,\hat{\tilde{p}}-\frac{\big (\hat{\tilde{E}}-\hat{V}_{car}(x,t)\big )^2}{2mc^2}, \end{aligned}$$

where $\hat{p}=-i\hbar \,\partial _x$ and $\hat{E}= i\hbar \,\partial _t$ are the usual Schrödinger generators, while the Post–Carroll generators are (see Section “Carroll–Schrödinger inner product and generators” and Ref.^34^)

5 $$\begin{aligned} \hat{\tilde{p}}=+\,i\hbar \,\frac{\partial }{\partial x}, \qquad \hat{\tilde{E}}=-\,i\hbar \,\frac{\partial }{\partial t}. \end{aligned}$$

With these definitions, Eqs. (2)–(3) read

6 $$\begin{aligned} \hat{\mathscr {H}}\Psi =0,\qquad \hat{\mathscr {F}}\Psi =0. \end{aligned}$$

We ask for a relation between the potentials that makes the two equations compatible in the sense that each operator preserves the constraint subspace defined by the other. A standard sufficient condition is vanishing commutator on a common invariant core $\mathscr {D}$,

7 $$\begin{aligned} {[}\hat{\mathscr {H}},\hat{\mathscr {F}}]=0 \quad \text {on } \mathscr {D}. \end{aligned}$$

If $\psi \in \mathscr {D}$ and $\hat{\mathscr {F}}\psi =0$, then $\hat{\mathscr {F}}(\hat{\mathscr {H}}\psi )=\hat{\mathscr {H}}(\hat{\mathscr {F}}\psi )=0$, so $\hat{\mathscr {H}}$ maps $\ker \hat{\mathscr {F}}\cap \mathscr {D}$ into itself; the same argument with $\hat{\mathscr {H}}$ and $\hat{\mathscr {F}}$ swapped shows that $\hat{\mathscr {F}}$ preserves $\ker \hat{\mathscr {H}}\cap \mathscr {D}$. Thus the two equations admit a stable class of *shared solutions* (but this does not imply $\ker \hat{\mathscr {H}}=\ker \hat{\mathscr {F}}$).

For the operators above, a direct computation of the commutator shows that the only way (7) can hold is if and only if

8 $$\begin{aligned} \partial _x V_{sch}=\partial _x V_{car}=0,\qquad \partial _t V_{sch}=\,-\partial _t V_{car}, \end{aligned}$$

i.e.

9 $$\begin{aligned} \boxed {\; V_{sch}(x,t)=V(t),\qquad V_{car}(x,t)=\,-V(t)+C \;(C\in \mathbb {R})\; }. \end{aligned}$$

Under (9) we have $[\hat{\mathscr {H}},\hat{\mathscr {F}}]=0$ on a common invariant core, so $\hat{\mathscr {H}}$ maps $\ker \hat{\mathscr {F}}$ into itself and vice versa.

One may then ask whether every separated Schrödinger solution for $V_{sch}=V_{sch}(t)$ also solve the Carroll–Schrödinger equation with the *same* wavefunction. Let $\Psi (x,t)=\phi _k(x)T_k(t)$ with

10 $$\begin{aligned} -\frac{\hbar ^2}{2m}\phi _k''=E_k\phi _k,\qquad i\hbar \,\dot{T}_k=(E_k+V_{sch}(t))\,T_k. \end{aligned}$$

Substituting this into the Post–Carroll operator and using $\hat{\tilde{E}}=-i\hbar \partial _t$,

11 $$\begin{aligned} {[}\hat{\tilde{E}}-\hat{V}_{car}(t)]\Psi =-\,[E_k+V_{sch}(t)+ V_{car}(t)]\Psi , \quad \Rightarrow \quad [\hat{\tilde{E}}-\hat{V}_{car}(t)]^{2}\Psi =[E_k+V_{sch} +V_{car}]^{2}\Psi . \end{aligned}$$

Then $$\hat{\mathscr {F}}\Psi =0$$ becomes

12 $$\begin{aligned} i\hbar c\,\frac{\phi _k'}{\phi _k} =\frac{[E_k+V_{sch}(t)+ V_{car}(t)]^{2}}{2mc^2}. \end{aligned}$$

The left side depends only on *x*, the right side only on *t*, so both must be time–independent; hence

13 $$\begin{aligned} \boxed {\ V_{car}(t)=-\,V_{sch}(t)+C\ }. \end{aligned}$$

In agreement with relation (9).

## From the time-independent Schrödinger equation to the space-independent Carroll–Schrödinger equation

Analogous to the derivation of the time–independent Schrödinger equation, a *space–independent* Carroll-Schrödinger equation follows from (3) when $V_{car}(x,t)= V_{car}(t)$. Dividing (3) by *c* and seeking separated states $\Psi (x,t)=\phi _x(x)\,\phi _t(t)$ gives

14 $$\begin{aligned} i\hbar \,\frac{\phi _x'}{\phi _x} =\frac{1}{2mc^{3}}\, \frac{\big (-i\hbar \,\partial _t- V_{car}(t)\big )^{2}\phi _t}{\phi _t} =:p_{0}, \qquad p_{0}\in \mathbb {R}, \end{aligned}$$

so that the spatial factor is a momentum eigenstate,

15 $$\begin{aligned} \phi _x(x)=\exp \!\Big (-\frac{i}{\hbar }p_{0}x\Big ), \qquad \hat{\tilde{p}}\,\phi _x=p_{0}\,\phi _x, \end{aligned}$$

and the time factor satisfies the *space–independent Carroll–Schrödinger equation*

16 $$\begin{aligned} \frac{1}{2mc^{3}}\Big (-i\hbar \,\frac{d}{dt}- V_{car}(t)\Big )^{2}\phi _t(t)-p_{0}\,\phi _t(t)=0. \end{aligned}$$

Multiplying (16) by *c* one obtains the energy form

17 $$\begin{aligned} \boxed {\;\frac{1}{2mc^{2}}\Big (-i\hbar \,\frac{d}{dt}- V_{car}(t)\Big )^{2}\phi _t(t)-cp_{0}\,\phi _t(t)=0.\;} \end{aligned}$$

Remembering that

18 $$\begin{aligned} cp_{0}=\frac{E_{0}^{2}}{2mc^{2}}, \qquad \text {so that}\quad E_{0}=\pm \sqrt{2mc^{3}p_{0}}, \end{aligned}$$

we may observe that the *defining* separation parameter is the Post–Carroll momentum $p_{0}$ (appearing linearly in (14–16); the energy label $E_{0}$ is not unique since $E_{0}$ and $-E_{0}$ correspond to the same $p_{0}$ via (18).

Having just derived the space–independent Carroll–Schrödinger Eq. (17), is it possible to find a coordinate transformation that will take us to the time–independent Schrödinger equation or vice versa?

19 $$\begin{aligned} -\frac{\hbar ^2}{2m} \frac{\partial ^2 \Psi _x}{\partial x^2} + {V_{sch}}(x) \Psi _x(x) = E_{sch} \Psi _x(x) \end{aligned}$$

Exploring a general case where *x* is an arbitrary function of *t*, we have

20 $$\begin{aligned} x = \delta (t), \quad \Psi (x) \rightarrow \Psi (\delta (t)) = \Phi (t) \end{aligned}$$

the time–independent Schrödinger equation becomes:

21 $$\begin{aligned} -\frac{\hbar ^2}{2m} \left[ \frac{\ddot{\Phi }}{\dot{\delta }^2} - \frac{\dot{\Phi } \ddot{\delta }}{\dot{\delta }^3}\right] + {V}_{sch}(\delta (t)) \Phi - E_{sch} \Phi = 0 \end{aligned}$$

Multiplying Eq. (21) by $\dot{\delta }^2/c^2$ and comparing with the expanded Carroll–Schrödinger equation, taking $\Psi _{car}(t)=\Phi (t)$,

22 $$\begin{aligned} -\frac{\hbar ^2}{2m c^2} \frac{\partial ^2 \Psi _{car}}{\partial t^2} + \frac{i\hbar {V}_{car} \dot{\Psi }_{car}}{m c^2} + \left[ \frac{i\hbar }{2m c^2} \frac{\partial {V}_{car}}{\partial t} + \frac{{V}^2 _{car} }{2m c^2} - cp_0\right] \Psi _{car} = 0 \end{aligned}$$

we obtain

23 $$\begin{aligned} \frac{\ddot{\delta }}{\dot{\delta }} = \frac{2i}{\hbar }V_{car}(t) \;\Rightarrow \; \delta (t) = C_1 + C_0 \int\limits^{t} \exp \!\left( \frac{2i}{\hbar }\int\limits^{t'} V_{car}(s)\,ds\right) dt' . \end{aligned}$$

(with any lower integration limits properly absorbed into the constants $C_0$ and $C_1$) and,

24 $$\begin{aligned} V_{sch}(x) = E_{sch} + \frac{1}{2m(\dot{\delta })^2}\left[ i\hbar \frac{dV_{car}(t)}{dt} + V_{car}^2(t) - E_{0}^2\right] _{t=\delta ^{-1}(x)} \end{aligned}$$

This is the transformation rule between the systems. Notably, the reparametrization $x=\delta (t)$ is potential dependent. We have found that, given a space–independent Carroll–Schrödinger equation with a specified potential, it may be transformed into a space–dependent Schrödinger equation under the map

25 $$\begin{aligned} t= & \delta ^{-1}(x), \quad \text {where} \quad x = \delta (t) = C_1 + C_0 \int\limits^{t} \exp \!\left( \frac{2i}{\hbar }\int\limits^{t'} V_{car}(s)\,ds\right) dt' \end{aligned}$$

26 $$\begin{aligned} V_{sch}(x)= & E_{sch} + \frac{1}{2m(\dot{\delta })^2}\left[ i\hbar \frac{dV_{car}(t)}{dt} + V_{car}^2(t) - E_{0}^2\right] _{t=\delta ^{-1}(x)} \end{aligned}$$

so that the solution of the Post–Carroll differential operator $\Phi _{car}(t)$ equals the solution of the equivalent Schrödinger operator $\Psi (x)$ evaluated at $x = \delta (t)$.

Alternatively, given a velocity profile of a given parametrization $v(t)=\dot{x}(t)=\dot{\delta }(t)$, an associated Post–Carroll potential is

$$\begin{aligned} V_{car}(t)= -\frac{i\hbar }{2} \frac{\dot{v}(t)}{v(t)}, \quad \text {and an associated dual Schr}\ddot{\textrm{o}}\text {dinger potential is given by} \end{aligned}$$

27 $$\begin{aligned} V_{sch}(x) = E_{sch} + \frac{1}{2m v(t)^2}\left[ \frac{\hbar ^2}{2} \frac{d}{dt}\left( \frac{\dot{v}(t)}{v(t)}\right) - \frac{\hbar ^2}{4}\left( \frac{\dot{v}(t)}{v(t)}\right) ^2 - E_{0}^2 \right] _{t=\delta ^{-1}(x)} \end{aligned}$$

### **Potential inversion procedure**

We begin from the reparametrization relations (with $x=\delta (t)$)

28 $$\begin{aligned} \frac{\ddot{\delta }}{\dot{\delta }}=\frac{2i}{\hbar }V_{\textrm{car}}(t), \qquad V_{\textrm{sch}}(\delta (t)) =E_{\textrm{sch}}+\frac{1}{2m\,\dot{\delta }(t)^{2}}\Big (i\hbar \,\dot{V}_{\textrm{car}}(t)+V_{\textrm{car}}(t)^{2}-E_{0}^{2}\Big ), \end{aligned}$$

Eliminating $V_{\textrm{car}}$ via $V_{\textrm{car}}= -\frac{i\hbar }{2}\frac{\ddot{\delta }}{\dot{\delta }}$ gives

29 $$\begin{aligned} V_{\textrm{sch}}(\delta (t))-E_{\textrm{sch}} =\frac{\hbar ^{2}}{4m}\,\frac{\{\delta ,t\}}{\dot{\delta }(t)^{2}} -\frac{E_{0}^{2}}{2m}\,\frac{1}{\dot{\delta }(t)^{2}}, \qquad \{\delta ,t\} :=\frac{\delta '''}{\delta '}-\frac{3}{2}\!\left( \frac{\delta ''}{\delta '}\right) ^{2}. \end{aligned}$$

where $\{\delta ,t\}$ is the Schwarzian derivative^37,38^. Writing $\tau (x):=\delta ^{-1}(x)$ and using the inversion identities $\big (\{\delta ,t\}/\dot{\delta }^{2}\big )\!\big |_{t=\tau (x)}=-\{\tau ,x\},\; \big (1/\dot{\delta }^{2}\big )\!\big |_{t=\tau (x)}=\tau '(x)^{2},$ we obtain the inverse equation

30 $$\begin{aligned} \boxed {\; \{\tau ,x\}+\frac{2E_{0}^{2}}{\hbar ^{2}}\,\tau '(x)^{2} =-\,\frac{4m}{\hbar ^{2}}\Big (V_{\textrm{sch}}(x)-E_{\textrm{sch}}\Big ). \;} \end{aligned}$$

To linearize, the Schwarzian chain rule $\{f\!\circ \!\tau ,x\}=\{f,\tau \}\tau '^{2}+\{\tau ,x\}$ and pick *f* with constant Schwarzian $\{f,u\}=2(E_{0}/\hbar )^{2}$; an explicit choice is $f(u)=\tan \!\big (\tfrac{E_{0}}{\hbar }u\big )$. Define

31 $$\begin{aligned} \sigma (x):=f(\tau (x))=\tan \!\Big (\tfrac{E_{0}}{\hbar }\,\tau (x)\Big ), \end{aligned}$$

so that $\{\sigma ,x\}=\{\tau ,x\}+\tfrac{2E_{0}^{2}}{\hbar ^{2}}\tau '^{2}$, and (30) becomes the pure Schwarzian equation

32 $$\begin{aligned} \boxed {\;\{\sigma ,x\}=-\,\frac{4m}{\hbar ^{2}}\Big (V_{\textrm{sch}}(x)-E_{\textrm{sch}}\Big ).\;} \end{aligned}$$

A standard construction then solves (32): if $y_{1},y_{2}$ form a fundamental system of

33 $$\begin{aligned} y''(x)+q(x)\,y(x)=0, \qquad q(x):=\frac{2m}{\hbar ^{2}}\Big (V_{\textrm{sch}}(x)-E_{\textrm{sch}}\Big ), \end{aligned}$$

then $\sigma (x)=y_{1}(x)/y_{2}(x)$ satisfies $\{\sigma ,x\}=-2q(x)$, hence (32). Therefore

34 $$\begin{aligned} \sigma (x)=\frac{y_{1}(x)}{y_{2}(x)}, \qquad \tau (x)=\frac{\hbar }{E_{0}}\arctan \!\big (\sigma (x)\big ), \qquad \boxed {\,\delta (t)=\tau ^{-1}(t)\,} \end{aligned}$$

(on any interval where $\tau$ is monotone). Substituting (34) into (30) and inverting reproduces (29), hence (28); this verifies that the constructed $\delta$ indeed yields precisely the target $V_{\textrm{sch}}$ for the chosen $E_{0}$ (equivalently $p_{0}$).

### Specific targets

We now record the corresponding linear ODE (33), a convenient fundamental pair $(y_{1},y_{2})$, and the resulting $\delta$ for three canonical static potentials.

*Harmonic oscillator:* $V_{\textrm{sch}}(x)=\tfrac{1}{2}m\omega ^{2}(x-x_{0})^{2}$. With $\xi =\sqrt{\tfrac{m\omega }{\hbar }}(x-x_{0})$, $z=\sqrt{2}\,\xi$, and $\nu =\tfrac{E_{\textrm{sch}}}{\hbar \omega }-\tfrac{1}{2}$, the ODE (33) is the parabolic–cylinder equation

35 $$\begin{aligned} \frac{d^{2}y}{dz^{2}}+\Big (\nu +\tfrac{1}{2}-\frac{z^{2}}{4}\Big )y=0. \end{aligned}$$

A fundamental pair is $y_{1}(x)=D_{\nu }(z)$, $y_{2}(x)=D_{\nu }(-z)$. Hence

36 $$\begin{aligned} \sigma (x)=\frac{D_{\nu }\!\big (\sqrt{2}\,\xi \big )}{D_{\nu }\!\big (-\sqrt{2}\,\xi \big )},\qquad \tau (x)=\frac{\hbar }{E_{0}}\arctan \!\big (\sigma (x)\big ),\qquad \delta (t)=\tau ^{-1}(t), \end{aligned}$$

which, by the construction above, produces *exactly* $V_{\textrm{sch}}(x)=\tfrac{1}{2}m\omega ^{2}(x-x_{0})^{2}$.

*Coulomb–like:*

37 $$\begin{aligned} V_{\textrm{sch}}(x) = \frac{\alpha }{x - x_{0}} \quad \text {(on an interval } x \gtrless x_{0}\text {).} \end{aligned}$$

Set

38 $$\begin{aligned} k = \frac{\sqrt{2m|E_{\textrm{sch}}|}}{\hbar }, \quad z = 2k(x - x_{0}), \quad \kappa = \frac{m\alpha }{\hbar ^{2}k}. \end{aligned}$$

Then (33) becomes the Whittaker normal form with $\mu = \tfrac{1}{2}$.

39 $$\begin{aligned} \frac{d^{2}Y}{dz^{2}}+\left( -\frac{1}{4}+\frac{\kappa }{z}+\frac{1/4-\mu ^{2}}{z^{2}}\right) Y=0, \qquad \mu =\frac{1}{2}\;\Rightarrow \;\frac{1}{4}-\mu ^{2}=0, \end{aligned}$$

i.e.

40 $$\begin{aligned} Y_{zz}+\left( -\frac{1}{4}+\frac{\kappa }{z}\right) Y=0. \end{aligned}$$

A fundamental pair is $y_{1}(x)=M_{\kappa ,\frac{1}{2}}\!\big (2k(x-x_{0})\big )$, $y_{2}(x)=W_{\kappa ,\frac{1}{2}}\!\big (2k(x-x_{0})\big )$. Thus

41 $$\begin{aligned} \sigma (x)=\frac{M_{\kappa ,\frac{1}{2}}\!\big (2k(x-x_{0})\big )}{W_{\kappa ,\frac{1}{2}}\!\big (2k(x-x_{0})\big )}, \qquad \tau (x)=\frac{\hbar }{E_{0}}\arctan \!\big (\sigma (x)\big ), \qquad \delta (t)=\tau ^{-1}(t), \end{aligned}$$

which yields *exactly* $V_{\textrm{sch}}(x)=\alpha /(x-x_{0})$.

*Free case:* $V_{\textrm{sch}}(x)\equiv 0$. Taking $E_{\textrm{sch}}=0$ gives $q\equiv 0$, so a fundamental pair is $y_{1}(x)=1$, $y_{2}(x)=x-x_{0}$. Hence $\sigma (x)=1/(x-x_{0})$, $\tau (x)=\tfrac{\hbar }{E_{0}}\arctan \!\big (\tfrac{1}{x-x_{0}}\big )$, and $\delta =\tau ^{-1}$, for which (30) (equivalently (29)) yields $V_{\textrm{sch}}\equiv 0$.

*Remarks.* (i) The construction applies on any subinterval where $y_{2}$ has no zeros so that $\tau$ is monotone and invertible; different branches correspond to different coordinate patches. (ii) Regarding the hermiticity of $V_{\textrm{car}}$ and the phase choice, from (28) one has

42 $$\begin{aligned} V_{\textrm{car}}(t) =-\,\frac{i\hbar }{2}\,\frac{\ddot{\delta }(t)}{\dot{\delta }(t)} =-\,\frac{i\hbar }{2}\,u(t), \qquad u(t):=\frac{\ddot{\delta }}{\dot{\delta }}. \end{aligned}$$

Requiring a *Hermitian* (real–valued) $V_{\textrm{car}}$ is therefore equivalent to demanding

43 $$\begin{aligned} u(t)\in i\mathbb {R}\quad \Longleftrightarrow \quad \frac{\ddot{\delta }}{\dot{\delta }}\ \text {is purely imaginary}. \end{aligned}$$

Writing $\tau =\delta ^{-1}$, the inverse–function relations give $\frac{\ddot{\delta }}{\dot{\delta }}=-\frac{\tau ''}{\tau '^2}.$ Hence a sufficient (and convenient) way to enforce (43) is to pick a branch with $\tau '(x)\in i\mathbb {R}$ on the *x*–interval of interest. This is achieved by the constant rescaling (for $|\sigma (x)| \ne 1$)

44 $$\begin{aligned} \sigma (x)\ \longrightarrow \ \sigma _H(x):=i\,\sigma (x), \quad \tau _H(x):=\frac{\hbar }{E_0}\arctan \big (\sigma _H(x)\big ) = i\,\frac{\hbar }{E_0}\operatorname {artanh}\!\big (\sigma (x)\big ), \quad \delta _H:=\tau _H^{-1}. \end{aligned}$$

Because the Schwarzian is invariant under constant Möbius rescalings, $\{\sigma _H,x\}=\{\sigma ,x\},$ (32) and all reconstruction formulas for $V_{\textrm{sch}}$ remain unchanged. In general, to ensure the hermiticity of the potentials (which would then require a complex map), the transformations already found need only be rescaled by the factor shown above.

## Probability currents and densities

It is well known that for the Schrödinger equation the probability density and current are

45 $$\begin{aligned} \rho _{sch} = \Psi _{sch}^* \Psi _{sch} , \quad J^x_{sch} = \frac{i\hbar }{2m}\!\left( \Psi _{sch} \frac{\partial \Psi ^* _{sch}}{\partial x} - \Psi ^* _{sch} \frac{\partial \Psi _{sch}}{\partial x}\right) , \end{aligned}$$

satisfying the continuity equation $\partial _t \rho + \partial _x J_x = 0$.

For the Carroll–Schrödinger case, a straightforward calculation gives

46 $$\begin{aligned} J^x_{car} = \Psi ^* _{car} \Psi _{car},\qquad \rho _{car} = \frac{-i\hbar }{2m c^3} \left( \Psi ^* _{car} \frac{\partial \Psi _{car}}{\partial t} - \Psi _{car}\frac{\partial \Psi ^* _{car}}{\partial t} \right) - \frac{\Psi ^* _{car} \Psi _{car} V_{car}}{m c^3}. \end{aligned}$$

Thus, spatial and temporal roles effectively interchange relative to the ordinary Schrödinger case; a notable feature is that the Post–Carroll density is potential dependent.

Again, we may relate these two results under a certain transformation. In $d=1+1$ with $J^\mu =(\rho ,\textbf{J})$,

47 $$\begin{aligned} J^\mu _{carroll}= & \left( \frac{-i\hbar }{2m c^3} \left( \Psi ^* _{car} \frac{\partial \Psi _{car}}{\partial t} - \Psi _{car}\frac{\partial \Psi ^* _{car}}{\partial t} \right) - \frac{\Psi ^* _{car}\Psi _{car} V_{car}(x,t)}{m c^3}, \; \Psi ^* _{car} \Psi _{car} \right) , \end{aligned}$$

48 $$\begin{aligned} J^\mu _{sch}= & \left( \Psi ^* _{sch}\Psi _{sch}, \; \frac{i\hbar }{2m}\left[ \Psi _{sch}\frac{\partial \Psi ^* _{sch}}{\partial x} - \Psi ^* _{sch} \frac{\partial \Psi _{sch}}{\partial x} \right] \right) . \end{aligned}$$

First, removing the explicit $V_{car}(x,t)$ from 47 by performing a gauge transformation of the form

49 $$\begin{aligned} \Phi _{car}(x,t)=\exp \!\left[ -\frac{i}{\hbar }\int ^{t}V_{car}(x,\tau )\,d\tau \right] \Psi _{car}(x,t), \end{aligned}$$

so that

50 $$\begin{aligned} \rho _{car}=\frac{-i\hbar }{2mc^{3}}\!\left( \Phi ^{*}_{car}\partial _{t}\Phi _{car}-\Phi _{car}\,\partial _{t} \Phi ^{*}_{car}\right) , \qquad J^{x}_{car}=|\Phi _{car}|^{2}. \end{aligned}$$

Now, performing a coordinate inversion of the form

51 $$\begin{aligned} x'=ct,\qquad t'=\,\frac{x}{c}, \end{aligned}$$

The Carroll continuity equation $\partial _{x}\rho _{car}+\partial _{t}J^{x}_{car}=0$ becomes exactly the Schrödinger continuity equation

52 $$\begin{aligned} \partial _{t'}|\Phi _{car}|^{2} +\partial _{x'}\!\left( \frac{i\hbar }{2m}\big [\Phi _{car}\,\partial _{x'}\Phi _{car}^{*}-\Phi _{car}^{*}\partial _{x'}\Phi _{car}\big ]\right) =0, \end{aligned}$$

As expected from the isomorphism between the Carroll-Schrödinger and Schrödinger algebras in 1+1 dimensions^34^.

## From Schrödinger to Carroll–Schrödinger in d=1+1 via a v$\rightarrow \infty$ boost

From Schrödinger to Carroll–Schrödinger in $d=1+1$ via a $v\!\rightarrow \!\infty$ boost

In $d=1{+}1$ the energy–momentum two–vector is $p^\mu =(E/c,\,P)$ and transforms under a standard Lorentz boost with speed *v* (along $+x$) as

53 $$\begin{aligned} \frac{E'}{c}=\gamma \!\left( \frac{E}{c}-\beta P\right) ,\qquad P'=\gamma \!\left( P-\beta \,\frac{E}{c}\right) , \qquad \beta :=\frac{v}{c},\ \ \gamma :=\frac{1}{\sqrt{1-\beta ^{2}}}. \end{aligned}$$

To evaluate the formal ultra–boost $V\rightarrow \infty$, the Lorentz formulas are analytically continued to $|\beta |>1$ by choosing the branch

54 $$\begin{aligned} \gamma =\frac{i}{\sqrt{\beta ^{2}-1}}\quad (|\beta |>1), \qquad \gamma \sim \frac{i}{\beta }\ \ \text {as}\ \ \beta \rightarrow \infty . \end{aligned}$$

Substituting (54) into (53) yields the ultra–boosted limits

55 $$\begin{aligned} \frac{E'}{c}=\gamma \!\left( \frac{E}{c}-\beta P\right) \xrightarrow [\beta \rightarrow \infty ]{}-\,i\,P, \qquad P'=\gamma \!\left( P-\beta \frac{E}{c}\right) \xrightarrow [\beta \rightarrow \infty ]{}-\,\frac{i}{c}\,E, \end{aligned}$$

i.e.

56 $$\begin{aligned} \boxed {\,E'=-\,i\,c\,P,\qquad P'=-\,\frac{i}{c}\,E\,}. \end{aligned}$$

On the chosen branch the invariant $p^\mu p_\mu =(E/c)^2-P^2$ is preserved.

The free Schrödinger energy–momentum relation,

57 $$\begin{aligned} E=\frac{P^{2}}{2m}\qquad \Longleftrightarrow \qquad E'=\frac{P'^2}{2m}, \end{aligned}$$

combined with (56) gives

58 $$\begin{aligned} \frac{(-iE/c)^2}{2m}=-\,i\,c\,P \ \Longrightarrow \ -\,\frac{E^{2}}{2m c^{2}}=-\,i\,c\,P \ \Longrightarrow \ \boxed {\,\frac{E^{2}}{2m c^{3}}=i\,P\,}. \end{aligned}$$

Observing the relation, we notice that the Post–Carroll energy–momentum relation is recovered under the mass redefinition $m\mapsto i m$, as in^32,34^.

## Hamilton–Jacobi limit

Starting from the free Carroll–Schrödinger Eq. (1)

59 $$\begin{aligned} i\hbar c\,\partial _x \Psi (x,t) + \frac{\hbar ^2}{2mc^2}\,\partial _t^2 \Psi (x,t)=0, \end{aligned}$$

we use the Wentzel–Kramers–Brillouin (WKB) *ansatz*

60 $$\begin{aligned} \Psi (x,t)=A(x,t)\,e^{\frac{i}{\hbar }S(x,t)} . \end{aligned}$$

Substituting into (59) and grouping real and imaginary terms yields the two equations

61 $$\begin{aligned} & \frac{\hbar }{2mc^{3}}\frac{\partial ^{2}A}{\partial t^{2}} -\frac{A}{2mc^{3}\hbar }\Bigl (\frac{\partial S}{\partial t}\Bigr )^{2} -\frac{A}{\hbar }\frac{\partial S}{\partial x} = 0 \end{aligned}$$

62 $$\begin{aligned} & \frac{1}{mc^{3}}\frac{\partial A}{\partial t}\frac{\partial S}{\partial t} +\frac{\partial A}{\partial x} = 0 \end{aligned}$$

Multiplying 61 by $\hbar$ and taking the classical limit $\hbar \rightarrow 0$ (discarding higher-order terms in $\hbar$), we obtain the associated Hamilton–Jacobi equation

63 $$\begin{aligned} \boxed {\; \frac{1}{2mc^{3}}\Bigl (\frac{\partial S}{\partial t}\Bigr )^{2} +\frac{\partial S}{\partial x}=0 \;} \end{aligned}$$

for the action *S*(*x*, *t*).

We may solve for the action using a separable ansatz of the form

64 $$\begin{aligned} S(x,t)=S_x(x)+S_t(t), \end{aligned}$$

which yields from the Hamilton–Jacobi equation

65 $$\begin{aligned} \frac{1}{2 m c^{3}}\!\left( \frac{\partial S}{\partial t}\right) ^{2} +\left( \frac{\partial S}{\partial x}\right) =0 \quad \Longrightarrow \quad \frac{1}{2 m c^{3}}\!\left( \frac{dS_t}{dt}\right) ^{2} +\left( \frac{dS_x}{dx}\right) =0 . \end{aligned}$$

By separation of variables we set

66 $$\begin{aligned} \frac{1}{2 m c^{3}}\!\left( \frac{dS_t}{dt}\right) ^{2}=\alpha , \qquad \left( \frac{dS_x}{dx}\right) =-\alpha , \end{aligned}$$

and therefore

67 $$\begin{aligned} S(x,t)=-\alpha \,x \pm \sqrt{2 m c^{3}\alpha }\; t + C_{0}. \end{aligned}$$

Here $\alpha$ is the separation constant with units of momentum; we relabel it as $p_{0}$ since it is a constant of motion. (*Sign convention:* The negative sign arises because in our Carroll representation the momentum operator is $\hat{p}_x=i\hbar \,\partial _x$ (cf.^32^). Acting on a (WKB) wave $\Psi =A\,e^{iS/\hbar }$ gives $\hat{p}_x\Psi =(i\hbar \,\partial _xA - A\,\partial _x S)\,e^{iS/\hbar }$; in the WKB limit the $i\hbar \,\partial _xA$ term is subleading, so $\hat{p}_x\Psi \approx (-\partial _x S)\Psi$, hence $p_x=-\partial _x S$).

The second constant follows from

68 $$\begin{aligned} \frac{\partial S}{\partial \alpha _i}=\beta _i \qquad \Longrightarrow \qquad \frac{\partial S}{\partial p_{0}} =-x \pm \sqrt{\frac{m c^{3}}{2p_{0}}}\; t=\beta _{\pm }. \end{aligned}$$

We observe that, from the Post–Carrollian dispersion relation,

69 $$\begin{aligned} E(p)= \pm \sqrt{2 m c^{3}p} \quad \Rightarrow \quad \frac{d {E}}{dp} = \pm \sqrt{\frac{m c^{3}}{2p}} =v_{\text {group}} . \end{aligned}$$

Thus, in the classical limit,

70 $$\begin{aligned} v_{\text {particle}}=\pm \sqrt{\frac{m c^{3}}{2p}}, \end{aligned}$$

and Eq. (68) may be rewritten as

71 $$\begin{aligned} x=\pm v_{\text {group}}\,t-\beta _{\pm }, \end{aligned}$$

so $\beta _{\pm }$ can be identified with the particle’s initial position.

In the classical limit of the one-dimensional free-particle case, the motion is rectilinear and uniform, showing no fundamental difference from the Newtonian limit of the one-dimensional free Schrödinger equation. This equivalence is lost once interactions are introduced. Nevertheless, even in the simplest one-dimensional free case, a fundamental difference between the formalisms appears in the momentum–velocity relation. Inverting Eq. (68) and solving for the magnitude of the momentum, we obtain

72 $$\begin{aligned} v_{p}=\pm \sqrt{\frac{m c^{3}}{2p}} \qquad \Longrightarrow \qquad |p|=\frac{m c^{3}}{2 v^{2}}, \quad E(p)= \pm \sqrt{2 m c^{3}p} = \pm \frac{mc^3}{v} \end{aligned}$$

Consistent with Ref.^34^, this result shows an inverse Energy-Velocity and Momentum–Velocity relationship, in sharp contrast to the Newtonian case.

### Extended phase space

Since time is not the evolution parameter, the extended phase-space approach is natural for analyzing Carrollian classical systems (cf.^31^). With a Post–Carrollian potential $V_\textrm{car}(x,t)$ the Hamilton–Jacobi equation reads

73 $$\begin{aligned} \frac{1}{2mc^{3}}\Bigl (\partial _t S +V_\textrm{car}(x,t)\Bigr )^{2} +\partial _x S=0. \end{aligned}$$

Identifying the generalized momenta (our convention $\hat{p}_x=i\hbar \partial _x$ implies $p_x=-\partial _x S$),

74 $$\begin{aligned} p_x=-\partial _x S, \qquad p_t=\partial _t S, \end{aligned}$$

the constraint equation is

75 $$\begin{aligned} F(x,t;p_x,p_t):=-c\,p_x+\frac{1}{2mc^{2}}\big (p_t + V_\textrm{car}(x,t)\big )^{2}=0. \end{aligned}$$

Its characteristic system (dot $=\tfrac{d}{d\lambda }$) is

76 $$\begin{aligned} & \dot{x}=\partial _{p_x}F=-c,\qquad \dot{t}=\partial _{p_t}F=\frac{p_t +V_\textrm{car}}{mc^{2}}, \end{aligned}$$

77 $$\begin{aligned} & \dot{p}_x=-\partial _x F= -\frac{p_t+ V_\textrm{car}}{mc^{2}}\partial _x V_\textrm{car}, \qquad \dot{p}_t=-\partial _t F= -\frac{p_t+ V_\textrm{car}}{mc^{2}}\partial _t V_\textrm{car}. \end{aligned}$$

From (76) we have $\dot{x}=-c$. A convenient gauge choice is $\lambda =-x/c$, so that $d/d\lambda =-c\,d/dx$ and $\dot{x}=-c$ identically. Defining

78 $$\begin{aligned} q(x):=p_t(x)+ V_\textrm{car}\big (x,t(x)\big ). \end{aligned}$$

Then, using gauge-invariant *x*-slopes,

79 $$\begin{aligned} \boxed {\quad \frac{dt}{dx}=-\,\frac{q(x)}{mc^{3}},\qquad \frac{dq}{dx}=\,\partial _x V_\textrm{car}\big (x,t(x)\big ),\qquad p_x(x)=\,\frac{q(x)^{2}}{2mc^{3}}\quad } \end{aligned}$$

*t*(*x*) obeys

80 $$\begin{aligned} \boxed {\ \frac{d^{2}t}{dx^{2}}=-\,\frac{1}{mc^{3}}\, \partial _x V_\textrm{car}\big (x,t(x)\big )\ }. \end{aligned}$$

#### General quadratures

For initial data $t(x_0)=t_0$, $q(x_0)=q_0$,

81 $$\begin{aligned} q(x)=q_0+\int\limits_{x_0}^{x}\partial _x V_\textrm{car}\big (\xi ,t(\xi )\big )\,d\xi ,\qquad t(x)=t_0-\frac{1}{mc^{3}}\int\limits_{x_0}^{x} q(\eta )\,d\eta . \end{aligned}$$

This gives the Picard iteration

82 $$\begin{aligned} t^{(0)}(x)&=t_0-\frac{q_0}{mc^{3}}(x-x_0),\end{aligned}$$

83 $$\begin{aligned} q^{(n+1)}(x)&=q_0+\int\limits_{x_0}^{x}\partial _x V_\textrm{car}\!\left( \xi ,\,t^{(n)}(\xi )\right) d\xi ,\end{aligned}$$

84 $$\begin{aligned} t^{(n+1)}(x)&=t_0-\frac{1}{mc^{3}}\int\limits_{x_0}^{x}q^{(n+1)}(\eta )\,d\eta . \end{aligned}$$

To first order in weak spatial dependence (small $\partial _x V_\textrm{car}$),

85 $$\begin{aligned} t(x)\approx t_0-\frac{q_0}{mc^{3}}(x-x_0) -\frac{1}{mc^{3}}\int\limits_{x_0}^{x}\!\!\Bigg [\int\limits_{x_0}^{\eta } \partial _x V_\textrm{car}\!\left( \xi ,\,t_0-\frac{q_0}{mc^{3}}(\xi -x_0)\right) d\xi \Bigg ]d\eta . \end{aligned}$$

#### Exactly solvable cases

We now explore the solutions behavior of specific cases of interest such as,

(A) *Time-only potential *$V_\textrm{car}(t)$

Here $\partial _x V_\textrm{car}=0$, hence $q(x)\equiv q_0$, and

86 $$\begin{aligned} t(x)=t_0-\frac{q_0}{mc^{3}}(x-x_0),\qquad p_x=\,\frac{q_0^{2}}{2mc^{3}}. \end{aligned}$$

We may observe that, for time-dependent potentials, the classical trajectory of the particle is identical to the free-particle case: a straight line with constant velocity. In this setting the combination $q_0=p_t+V_\textrm{car}(t)$ is conserved, so $p_t=q_0 - V_\textrm{car}(t)$ varies in time;consequently, energy is not conserved, whereas the spatial momentum $p_x=\tfrac{q_0^{2}}{2mc^{3}}$ remains constant.

(B) *Space-only potential *$V_\textrm{car}(x)$

Then $p_t(x)\equiv p_{t,0}$ and $q(x)=p_{t,0}+V_\textrm{car}(x)$, so

87 $$\begin{aligned} t(x)=t_0-\frac{1}{mc^{3}}\int\limits_{x_0}^{x}\big (p_{t,0}+V_\textrm{car}(\xi )\big )\,d\xi ,\qquad p_x(x)=\,\frac{\big (p_{t,0}+V_\textrm{car}(x)\big )^{2}}{2mc^{3}}. \end{aligned}$$

Exploring two useful subcases corresponding to the most common classical potentials in the analog newtonian dynamics

88 $$\begin{aligned}&V_\textrm{car}(x)=V_0+\alpha x \ \Rightarrow \ t(x)=t_0-\frac{p_{t,0}+V_0}{mc^{3}}(x-x_0)-\frac{\alpha }{2mc^{3}}\big (x^{2}-x_0^{2}\big ),\end{aligned}$$

89 $$\begin{aligned}&V_\textrm{car}(x)=\tfrac{\kappa }{2}x^{2} \ \Rightarrow \ t(x)=t_0-\frac{p_{t,0}}{mc^{3}}(x-x_0)-\frac{\kappa }{6mc^{3}}\big (x^{3}-x_0^{3}\big ). \end{aligned}$$

It is important to note the strong departures from standard classical dynamics when potentials are introduced: space becomes the evolution parameter, and in this limit motion may proceed forward or backward in time.

For the space-only potential case, a natural question arises: is there a relationship between the spatial potentials $V_\textrm{sch}(x)$ and $V_\textrm{car}(x)$ such that the classical trajectories match? We address this for space-only potentials on intervals with no turning points ($\dot{x}=v\ne 0$), so that one may regard $t$ as a function of $x$.

The Newtonian and Post–Carrollian equations read

$$m\,\frac{d^{2}x}{dt^{2}}=-\,\frac{dV_\textrm{sch}}{dx}(x),\qquad m c^{3}\,\frac{d^{2}t}{dx^{2}}=-\,\frac{dV_\textrm{car}}{dx}(x).$$

For inverse functions one has the identity

90 $$\begin{aligned} \frac{d^{2}t}{dx^{2}} =-\,\frac{\ddot{x}}{(\dot{x})^{3}}, \end{aligned}$$

Eliminating $$\ddot{x}$$ gives

91 $$\begin{aligned} \boxed {\; \frac{dV_\textrm{car}}{dx}(x) =-\,\frac{c^{3}}{v(x)^{3}}\,\frac{dV_\textrm{sch}}{dx}(x)\; }. \end{aligned}$$

Along a Newtonian trajectory with conserved energy

$$E_{newton}=\tfrac{1}{2} m\,v(x)^{2}+V_\textrm{sch}(x) \quad \Rightarrow \quad v(x)=\sqrt{\frac{2}{m}\,\big (E_{newton}-V_\textrm{sch}(x)\big )},$$

(91) becomes

$$\frac{dV_\textrm{car}}{dx} =-\,\frac{m^{3/2}c^{3}}{(2)^{3/2}} \frac{1}{\big (E_{newton}-V_\textrm{sch}(x)\big )^{3/2}} \frac{dV_\textrm{sch}}{dx}.$$

Integrating with respect to $x$ (equivalently, with respect to $V_\textrm{sch}$) yields

92 $$\begin{aligned} \boxed {\; V_\textrm{car}(x) = C\;-\;\frac{m^{3/2}c^{3}}{\sqrt{2}}\, \frac{1}{\sqrt{\,E_{newton}-V_\textrm{sch}(x)\,}}\; }, \end{aligned}$$

where $C$ sets the reference level of $V_\textrm{car}$.

It is important to note that the map (92) holds *piecewise* on regions with no turning points, i.e. where $E>V_\textrm{sch}(x)$ and $\dot{x}=v(x)\ne 0$. At a turning point ($E=V_\textrm{sch}(x)$) the inverse slope $dt/dx$ diverges and the map becomes singular, so matching must be done between turning points.

## Carroll–Schrödinger inner product and generators

We now treat *x* as the evolution variable and, for each fixed *x*, view the wavefunction as a vector $\Psi _x(t):=\Psi (x,t)$ in the equal-*x* Hilbert space $\mathscr {H}:=L^2(\mathbb {R}_t,dt)$. With this viewpoint the free Carroll–Schrödinger equation

93 $$\begin{aligned} i\hbar c\,\partial _x\Psi (x,t)\;+\;\frac{\hbar ^2}{2mc^2}\,\partial _t^2\Psi (x,t)\;=\;0 \end{aligned}$$

reads naturally as a Schrödinger-type *x*-evolution,

94 $$\begin{aligned} i\hbar \,\partial _x\Psi (x,\cdot )=H_x\,\Psi (x,\cdot ), \qquad H_x:=\frac{\hbar ^2}{2mc^{3}}\big (-\partial _t^2\big ), \end{aligned}$$

so $H_x$ plays the role of the *x*-evolution generator on $\mathscr {H}$.

Before analyzing the operators, it is helpful to see directly that the *t*-inner product is conserved along *x*. Multiplying (93) by $\Psi ^*$ and subtracting the conjugate equation, we obtain the continuity law

95 $$\begin{aligned} \partial _x\rho _{t}+\partial _t J_{t}=0, \qquad \rho _{t}:=|\Psi |^2, \qquad J_{t}:=\frac{\hbar }{mc^3}\,\Im \!\big (\Psi ^*\partial _t\Psi \big ) =\frac{\hbar }{2i\,mc^3}\!\big (\Psi ^*\partial _t\Psi -\Psi \,\partial _t\Psi ^*\big ). \end{aligned}$$

(Naming $J_t$ and $\rho _t$ emphasizes their role with respect to the time direction and the inner product.) Now, under standard boundary hypotheses in *t* (e.g. $\Psi ,\partial _t\Psi \in L^2(\mathbb {R})$ with $J_t(x,t)\rightarrow 0$ as $t\rightarrow \pm \infty$, or periodic/quasi-periodic conditions), integration in *t* gives

96 $$\begin{aligned} \frac{d}{dx}\int\limits_{\mathbb {R}}|\Psi (x,t)|^2\,dt=0 \quad \Longrightarrow \quad \Vert \Psi (x,\cdot )\Vert _{\mathscr {H}}=\Vert \Psi (x_0,\cdot )\Vert _{\mathscr {H}}\ \ \text {for all }x,x_0, \end{aligned}$$

so the equal-*x* inner product $\langle \phi ,\psi \rangle _t:=\int _{\mathbb {R}}\phi (t)^*\psi (t)\,dt$ is conserved along the *x*-flow.

We next analyze the operators. To make self-adjointness transparent we pass to frequency space via the *unitary* Fourier transform

97 $$\begin{aligned} (\mathscr {F}\psi )(\omega ):=\frac{1}{\sqrt{2\pi }}\int\limits_{\mathbb {R}}e^{-i\omega t}\psi (t)\,dt, \qquad \mathscr {F}^{-1}\widetilde{\psi }(t)=\frac{1}{\sqrt{2\pi }}\int\limits_{\mathbb {R}}e^{+i\omega t}\widetilde{\psi }(\omega )\,d\omega . \end{aligned}$$

In this representation, $-\partial _t^2$ becomes multiplication by $\omega ^2$, hence it is self-adjoint on $H^2(\mathbb {R})$ (and essentially self-adjoint on $\mathscr {S}(\mathbb {R})$). Consequently,

98 $$\begin{aligned} H_x=\frac{\hbar ^2}{2mc^{3}}(-\partial _t^2) \quad \text {is self-adjoint on}\quad D(H_x)=H^2(\mathbb {R})\subset \mathscr {H}. \end{aligned}$$

Now, defining the energy operator *spectrally*, set

99 $$\begin{aligned} (\widehat{E}\,\widetilde{\psi })(\omega ):=\hbar \,\omega \,\widetilde{\psi }(\omega ), \qquad D(\widehat{E})=\Big \{\widetilde{\psi }\in L^2(\mathbb {R}_\omega ):\ \omega \,\widetilde{\psi }\in L^2(\mathbb {R}_\omega )\Big \}. \end{aligned}$$

Pulling back by the unitary Fourier transform, we obtain on the time side

100 $$\begin{aligned} \boxed {\ \widehat{E}=\mathscr {F}^{-1}(\hbar \,\omega )\mathscr {F}=-\,i\hbar \,\partial _t \ \ \text {with domain}\ \ D(\widehat{E})=H^1(\mathbb {R})\ }, \end{aligned}$$

and, under the *t*-inner product, $\widehat{E}$ is Hermitian: for $\phi ,\psi \in \mathscr {S}(\mathbb {R})$ (or in $H^1$ with the boundary hypotheses above),

101 $$\begin{aligned} \langle \phi ,\widehat{E}\psi \rangle _t-\langle \widehat{E}\phi ,\psi \rangle _t =-\,i\hbar \!\int\limits_{\mathbb {R}}\!\phi ^*(t)\,\partial _t\psi (t)\,dt +\,i\hbar \!\int\limits_{\mathbb {R}}\!(\partial _t\phi )^*(t)\,\psi (t)\,dt =-\,i\hbar \,[\phi ^*(t)\psi (t)]_{-\infty }^{+\infty }=0. \end{aligned}$$

Hence $\widehat{E}$ is self-adjoint on $H^1(\mathbb {R})$, and, spectrally, the *x*-generator is simply the quadratic function of $\widehat{E}$,

102 $$\begin{aligned} \boxed {\ H_x=\frac{\widehat{E}^{\,2}}{2mc^{3}}\ }. \end{aligned}$$

By Stone’s theorem the family $U_x(a):=\exp \!\big (-\tfrac{i}{\hbar }aH_x\big )$ is a strongly continuous unitary group on $\mathscr {H}$, and the *x*–evolution is given by

103 $$\begin{aligned} \Psi (x,\cdot )=U_x(x-x_0)\,\Psi (x_0,\cdot ), \end{aligned}$$

which is another way to read the conservation law (96).

Finally, to connect with the Hamilton–Jacobi discussion, we recall our momentum convention $\widehat{p}:=+\,i\hbar \,\partial _x$, which in WKB implies $p_x=-\partial _x S$. On *solutions* of (94) the differential generator coincides with $H_x$, namely $\widehat{p}\,\Psi =H_x\,\Psi$, and for sufficiently regular solutions $\Phi ,\Psi$ we therefore have

104 $$\begin{aligned} \langle \Phi ,\widehat{p}\,\Psi \rangle _t-\langle \widehat{p}\,\Phi ,\Psi \rangle _t =\langle \Phi ,H_x\Psi \rangle _t-\langle H_x\Phi ,\Psi \rangle _t=0, \end{aligned}$$

which is nothing but the bilinear version of the continuity Eq. (95). On the full line $\mathbb {R}_t$ no endpoint conditions are required for self-adjointness: $-i\hbar \,\partial _t$ is essentially self-adjoint on $C_c^\infty (\mathbb {R})$ with closure $H^1(\mathbb {R})$, and $H_x$ is self-adjoint on $H^2(\mathbb {R})$. On a finite time interval $[T_1,T_2]$, periodic/quasi-periodic or suitable separated boundary conditions ensure the vanishing of the *t*-boundary contribution in (101) and (95), after which the same argument carries through unchanged.

## Solutions analysis

### Gaussian wave packet

Consider the free Carroll–Schrödinger evolution

105 $$\begin{aligned} i\hbar c\,\partial _x \Psi (x,t) + \frac{\hbar ^2}{2mc^2}\,\partial _t^2 \Psi (x,t)=0, \end{aligned}$$

viewed as a Schrödinger-type equation in the evolution variable *x* on the Hilbert space $\mathscr {H}=L^2(\mathbb {R}_t,dt)$, with self-adjoint generator

106 $$\begin{aligned} i\hbar \,\partial _x\Psi =H_x\Psi , \qquad H_x=\frac{\hbar ^2}{2mc^3}\,(-\partial _t^2) \quad \text {on } H^2(\mathbb {R}_t). \end{aligned}$$

We take a normalized Gaussian in time at $x=0$,

107 $$\begin{aligned} \Psi (0,t)=\Psi _0(t):=\frac{1}{(\pi \sigma ^2)^{1/4}}\, \exp \!\left[ -\frac{(t-t_0)^2}{2\sigma ^2}\right] , \qquad \Vert \Psi _0\Vert _{L^2(\mathbb {R}_t)}=1, \end{aligned}$$

with temporal width $\sigma >0$ and center $t_0\in \mathbb {R}$.

Using the unitary Fourier transform in *t*,

108 $$\begin{aligned} \widetilde{\Psi }(x,\omega )=\frac{1}{\sqrt{2\pi }}\!\int\limits_{\mathbb {R}}e^{-i\omega t}\Psi (x,t)\,dt, \qquad \Psi (x,t)=\frac{1}{\sqrt{2\pi }}\!\int\limits_{\mathbb {R}}e^{+i\omega t}\widetilde{\Psi }(x,\omega )\,d\omega , \end{aligned}$$

we have $\partial _t^2\!\rightarrow -\omega ^2$, hence

109 $$\begin{aligned} \partial _x \widetilde{\Psi }(x,\omega ) =-\,i\,\beta \,\omega ^2\,\widetilde{\Psi }(x,\omega ), \qquad \beta :=\frac{\hbar }{2mc^3}, \end{aligned}$$

so *x*–evolution multiplies each frequency component by the pure phase $e^{-i\beta x\omega ^2}$. Evaluating the Gaussian integral gives the exact solution

110 $$\begin{aligned} \Psi (x,t) = \frac{1}{(\pi \sigma ^2)^{1/4}}\, \frac{1}{\sqrt{D(x)}}\, \exp \!\left[ -\,\frac{(t-t_0)^2}{2\sigma ^2\,D(x)}\right] , \qquad D(x):=1 + i\,\chi (x),\quad \chi (x):=\frac{\hbar x}{m c^3\,\sigma ^2}. \end{aligned}$$

The conserved density and current (from $\partial _x\rho _t+\partial _t J_t=0$, cf. Eq. 95) are

111 $$\begin{aligned} \rho _t(x,t)=|\Psi (x,t)|^2 =\frac{1}{\sqrt{\pi }\,\sigma \,\sqrt{1+\chi (x)^2}}\, \exp \!\left[ -\,\frac{(t-t_0)^2}{\sigma ^2\bigl (1+\chi (x)^2\bigr )}\right] , \end{aligned}$$

112 $$\begin{aligned} J_t(x,t) =\frac{\hbar }{mc^3}\,\Im \!\big (\Psi ^*\partial _t\Psi \big ) =\frac{\hbar }{mc^3}\, \frac{\chi (x)}{\sigma ^2\bigl (1+\chi (x)^2\bigr )}\,(t-t_0)\,\rho _t(x,t). \end{aligned}$$

In particular, the equal-*x* norm is preserved for all *x*; explicitly,

113 $$\begin{aligned} \int\limits_{\mathbb {R}}\rho _t(x,t)\,dt =\frac{1}{\sqrt{\pi }\,\sigma \sqrt{1+\chi (x)^2}} \int\limits_{\mathbb {R}}\exp \!\left[ -\frac{(t-t_0)^2}{\sigma ^2\bigl (1+\chi (x)^2\bigr )}\right] dt =1, \end{aligned}$$

by $\int _{\mathbb {R}}e^{-y^2/a^2}dy=\sqrt{\pi }\,a$. Physically, this parallels the usual Schrödinger packet with the roles of space and time exchanged: the packet remains centered at $t_0$ while its temporal width disperses as

114 $$\begin{aligned} \sigma _{\textrm{eff}}(x)=\sigma \,\sqrt{1+\chi (x)^2} =\sqrt{\sigma ^2 + \left( \frac{\hbar x}{m c^3\,\sigma }\right) ^2}, \end{aligned}$$

and $J_t(x,t)$ is odd about $t_0$, encoding the *t*-flux that balances the *x*-rate of change of $\rho _t$ in the continuity equation. Since $H_x$ is self-adjoint on $H^2(\mathbb {R}_t)$, the *x*–evolution is unitary on $L^2(\mathbb {R}_t)$, which implies (113) under the boundary conditions stated in the previous section.

Finally, if one adds a carrier phase to the initial datum,

115 $$\begin{aligned} \Psi _0(t)\ \longrightarrow \ \Psi _0(t)\,e^{-i\omega _0(t-t_0)} \quad (E_0=\hbar \omega _0), \end{aligned}$$

the solution acquires the same complex width *D*(*x*) but its center drifts. A stationary-phase estimate of the inverse-transform phase $\omega t-\beta x\omega ^2$ gives

116 $$\begin{aligned} t_{\mathrm c}(x)=t_0+\frac{E_0}{mc^3}\,x, \qquad \Rightarrow \qquad \frac{dt_{\mathrm c}}{dx}=\frac{E_0}{mc^3} =\frac{d\kappa }{d\omega }\bigg |_{\omega _0}, \end{aligned}$$

since the *x*–wavenumber is $\kappa (\omega )=\beta \omega ^2$. Thus $dx/dt_{\mathrm c}=mc^3/E_0$ equals the classical (ray) velocity obtained from the Hamilton–Jacobi/characteristics analysis (72), in perfect agreement with the dispersion picture.

### Finite–time perturbations for a purely time–dependent potential

We consider the $(1+1)$–dimensional equation

117 $$\begin{aligned} i\hbar c\,\partial _x \Psi (x,t) -\frac{1}{2 m c^{2}}\!\left( -\,i\hbar \,\partial _t - V(t)\right) ^{\!2}\Psi (x,t)=0, \end{aligned}$$

for a scalar wavefunction $\Psi (x,t)$ coupled to a time–dependent scalar potential *V*(*t*). Seeking separable solutions of the form

118 $$\begin{aligned} \Psi (x,t)=\psi _t(t)\,\psi _x(x), \end{aligned}$$

Eq. (117) yields

119 $$\begin{aligned} i\hbar c\,\frac{\psi _x'(x)}{\psi _x(x)} = \frac{1}{2 m c^{2}}\, \frac{\left( i\hbar \,\frac{d}{dt}+V(t)\right) ^{2}\psi _t(t)}{\psi _t(t)} = cp_0, \end{aligned}$$

The spatial factor solves $i\hbar c\,\psi _x'(x)=E_0^{2}\psi _x(x)$ and is therefore a plane wave,

120 $$\begin{aligned} \psi _x(x)=\exp \!\left( \frac{i p_0 x}{\hbar }\right) , \qquad \text {with } \; E_0^{2} = 2 m c^{3} p_0 , \end{aligned}$$

for some real constant $p_0$ fixed by boundary conditions. The temporal factor obeys

121 $$\begin{aligned} \left( i\hbar \,\frac{d}{dt}+V(t)\right) ^{2}\psi _t(t)=E_0^{2}\,\psi _t(t). \end{aligned}$$

A convenient gauge transformation removes the explicit potential from (121). Defining

122 $$\begin{aligned} \phi (t):=\exp \!\left( \frac{-i}{\hbar }\int\limits_{t_0}^{t} V(t')\,dt'\right) \psi _t(t). \end{aligned}$$

And using (122) in (121) gives the free second–order equation

123 $$\begin{aligned} \frac{d^{2}\phi }{dt^{2}}+\frac{E_0^{2}}{\hbar ^{2}}\,\phi (t)=0. \end{aligned}$$

Now, for illustrative purposes, suppose the perturbation acts only on a finite interval $t\in [0,T]$. Consistency with compact support in time enforces the homogeneous boundary conditions

124 $$\begin{aligned} \phi (0)=\phi (T)=0. \end{aligned}$$

Equations (123)–(124) admit the discrete family of solutions

125 $$\begin{aligned} \phi _n(t)=A\,\sin \!\left( \frac{E_n t}{\hbar }\right) , \qquad E_n=\frac{n\pi \hbar }{T}, \quad n\in \mathbb {N}. \end{aligned}$$

Thus, restricting the interaction to a finite temporal interval leads to an energy quantization $E_n=n\pi \hbar /T$, in direct analogy with the spatial quantization for a particle in a finite box in the Schrödinger problem.

Imposing unit normalization in time over the active window,

126 $$\begin{aligned} \int\limits_{0}^{T}\! |\Psi (x,t)|^{2}\,dt = 1, \end{aligned}$$

fixes the amplitude in (125) to

127 $$\begin{aligned} A=\sqrt{\frac{2}{T}}. \end{aligned}$$

Combining (122), (120), and (127), the normalized modes read

128 $$\begin{aligned} \Psi _{n}(x,t) = \exp \!\left( \frac{i p_0 x}{\hbar }\right) \exp \!\left( \frac{i}{\hbar }\int\limits_{t_0}^{t} V(t')\,dt'\right) \sqrt{\frac{2}{T}}\, \sin \!\left( \frac{n\pi t}{T}\right) , \quad 0\le t\le T . \end{aligned}$$

Because the gauge factor in (128) has unit modulus, the probability density is independent of *V*(*t*),

129 $$\begin{aligned} \rho _{n}(x,t):=|\Psi _{n}(x,t)|^{2}=\frac{2}{T}\,\sin ^{2}\!\left( \frac{n\pi t}{T}\right) . \end{aligned}$$

Analyzing the temporal current, for the present spatially stationary state, the temporal component of the conserved current reduces to two equal and opposite contributions proportional to *V*(*t*), namely

130 $$\begin{aligned} J_{t}(t) = -\,\frac{V(t)}{m c^{2}}\,\rho _{n}(x,t) + \frac{V(t)}{m c^{2}}\,\rho _{n}(x,t) = 0, \end{aligned}$$

so that the temporal current vanishes identically while the density (129) remains unaffected by the detailed time profile of the potential.

### General space–time dependent potential

We now allow the interaction to depend on both space and time, $V=V(x,t)$, and consider the equation

131 $$\begin{aligned} i\hbar c\,\partial _x \Psi (x,t) -\frac{1}{2 m c^{2}}\!\left( -\,i\hbar \,\partial _t - V(x,t)\right) ^{\!2}\Psi (x,t)=0 . \end{aligned}$$

Introducing the gauge transform

132 $$\begin{aligned} \phi (x,t) := \exp \!\left( \frac{-i}{\hbar }\int\limits_{t_0}^{t} V(x,\tau )\,d\tau \right) \Psi (x,t) , \end{aligned}$$

a direct computation shows that the potential cancels from the second-order time operator, while the spatial derivative acquires a memory term

133 $$\begin{aligned} \partial _t\phi =\exp \!\left( \tfrac{-i}{\hbar }\!\int\limits_{t_0}^{t}\!V\,d\tau \right) \partial _t\Psi -\frac{i}{\hbar }V(x,t)\,\phi , \quad \partial _x\phi =\exp \!\left( \tfrac{-i}{\hbar }\!\int\limits_{t_0}^{t}\!V\,d\tau \right) \partial _x\Psi -\frac{i}{\hbar }\Bigg (\int\limits_{t_0}^{t}\!\partial _x V(x,\tau )\,d\tau \Bigg )\phi . \end{aligned}$$

Substituting into (131) yields the reduced equation

134 $$\begin{aligned} i\hbar c\,\partial _x \phi (x,t) + \frac{\hbar ^{2}}{2 m c^{2}}\,\partial _{t}^{2}\phi (x,t) - c\Bigg (\int\limits_{t_0}^{t}\partial _x V(x,\tau )\,d\tau \Bigg )\phi (x,t)=0 . \end{aligned}$$

Equivalently, isolating the *x*-evolution gives a Schrödinger–type equation with *x* playing the role of “time”,

135 $$\begin{aligned} i\hbar c\,\partial _x \phi (x,t) = -\,\frac{\hbar ^{2}}{2 m c^{2}}\,\partial _{t}^{2}\phi (x,t) + c\,F(x,t)\,\phi (x,t), \qquad F(x,t):=\int\limits_{t_0}^{t}\partial _x V(x,\tau )\,d\tau . \end{aligned}$$

Since the transformation (132) is unitary, one has $|\phi |^{2}=|\Psi |^{2}$.

Analyzing Eq. (135), we note that the gauge-transformed equation can also be obtained by viewing the interaction as entering through the momentum. In particular, to include an interacting case (analogous to a scalar potential in the Schrödinger equation), it is equivalent to implement a minimal substitution on the momentum,

136 $$\begin{aligned} p \;\longmapsto \; p' = p - F(x,t), \end{aligned}$$

where *F*(*x*, *t*) denotes the interaction momentum, in direct analogy with the standard energy shift $E\mapsto E' = E - V(x,t)$ in nonrelativistic quantum mechanics. This is consistent with the Post–Carrollian viewpoint in which dynamics is governed by the momentum sector rather than by the energy.

It is worth noting that Eq. 135 has a precise structural similarity to the equations describing the evolution of temporal solitons^35,36^. This is important because it suggests that temporal solitons can be regarded as inherently exhibiting Post–Carroll symmetry.

Now, in order to solve Eq. 135 for a general space–time interaction, we proceed with perturbation theory, splitting the potential into a solvable time–dependent part plus a small space–dependent perturbation:

137 $$\begin{aligned} c\,f(x,t) \;=\; g(t) \;+\; \varepsilon \,\eta (x), \qquad 0<\varepsilon \ll 1 . \end{aligned}$$

Define the unperturbed *x*-evolution operator $\mathscr {U}_{0}(x,x_{0})$ through

138 $$\begin{aligned} i\hbar c\,\partial _x \mathscr {U}_{0}(x,x_{0}) = \Big [-\,\tfrac{\hbar ^{2}}{2 m c^{2}}\,\partial _{t}^{2} - g(t)\Big ] \mathscr {U}_{0}(x,x_{0}), \qquad \mathscr {U}_{0}(x_{0},x_{0})=\textbf{1}. \end{aligned}$$

(When *g*(*t*) alone is present, the modes coincide with those constructed in Sec. 7 after the unitary gauge reduction.) The full solution admits a Dyson expansion in the “spatial time” *x*:

139 $$\begin{aligned} \phi (x,t) = \mathscr {U}_{0}(x,x_{0})\,\phi (x_{0},t) -\frac{i\,\varepsilon }{\hbar c}\int\limits_{x_{0}}^{x} \mathscr {U}_{0}(x,\xi )\,\eta (\xi )\,\mathscr {U}_{0}(\xi ,x_{0})\, \phi (x_{0},t)\,d\xi \;+\; \mathscr {O}(\varepsilon ^{2}). \end{aligned}$$

Equations (135)–(139) provide a consistent and systematic framework to treat general interactions *V*(*x*, *t*): the gauge transform removes the explicit *V* from the temporal operator, converts the problem into Schrödinger–type evolution in *x*, and relates the usual scalar potential to an *interaction momentum* *F*(*x*, *t*) via (136).

## Discussion

In $1{+}1$ dimensions, the free Carroll–Schrödinger structure is mathematically equivalent to the standard Schrödinger structure under the inversion $x \leftrightarrow ct$, reflecting the corresponding group isomorphism. The results of this paper therefore emphasized the genuinely new content that appears once one allows interactions: while the free-generator map is essentially kinematic, external potentials produce nontrivial operator-level compatibility constraints, nontrivial dualities between interacting problems, and a practical translation framework built around gauge reductions, reparametrizations, and controlled spatial Dyson expansions. It is important to distinguish these results from the results of *standard* Carroll-invariant quantum equations. Classic results show that a Carroll equation compatible with Carroll causality can only involve temporal derivatives of the fields^39^. Moreover, geometric quantization indicates that the resulting Carroll quantum equation (in the absence of electromagnetic interactions) is trivial^40^. For this reason, Eq. (1) is not strictly Carrollian in the standard sense, and both it and its higher-dimensional generalizations are more naturally understood within the *post–Carrollian* regime, as mentioned throughout the manuscript and also emphasized in^34^.

The framework developed here suggests several natural extensions. A first direction is to test which parts of the operator-level dictionary survive in higher dimensions and, in particular, whether the potential-dependent map $x=\delta (t)$ admits a meaningful multidimensional analogue. Beyond kinematics, one should revisit the equal-*x* Hilbert-space picture: the natural choice $L^{2}(\mathbb {R}^{d}_{t},dt)$ remains available, provided there is still a single time dimension in higher-dimensional generalizations and the equation remains first order in spatial derivatives. However, the specifics of the formalism may vary with the structure of the generalized equation.

There already exist nontrivial three-dimensional generalizations of post–Carrollian quantum dynamics with distinct structural choices and symmetry inputs. Comparing our derivations with those setups would help isolate which features are universal. For example, recent proposals for three-dimensional post–Carrollian quantum dynamics^32,34,41^ suggest alternative expressions for generalized 3D equations; importantly, both treat time as one dimensional and space as three dimensional. It would be natural to investigate: (i) the behavior of solutions to the three-dimensional equations for both single- and multi-particle systems; (ii) how the interaction-momentum picture extends to a vector field $\textbf{f}(x,t)$ in higher dimensions and its physical interpretation in contrast with ordinary potential energy; (iii) how the gauge/inversion map relating continuity equations changes in view of the space–time asymmetry in higher dimensions; and (iv) whether there exists an expansion for Klein–Gordon solutions in terms of Schrödinger and Post–Carroll–Schrödinger solutions.

Finally, explorations of quantum-field-theoretic interactions and fermionic extensions of post–Carrollian equations provide fertile ground for further study of Post–Carrollian solutions and their implications. We note that while the present study is theoretical, empirical validation of Carrollian and post–Carrollian structures may arise in condensed-matter physics—for example, in quantum systems possessing post–Carrollian symmetry, including analyses of temporal solitons, graphene and Carrollian fluids^30^—and in gravitational theory, particularly in view of proposed connections between Carroll fluids/particles and dark-energy and dark-matter components^31,34^, as well as recent developments in post–Carrollian gravity^33^.

## Data Availability

All data supporting the findings of this study are available within the paper. This is a theoretical study and does not contain experimental data.
